# 3-Methyl-1,2,4-triazolo[3,4-*a*]phthalazine monohydrate

**DOI:** 10.1107/S1600536809040677

**Published:** 2009-10-10

**Authors:** Grzegorz Dutkiewicz, C. S. Chidan Kumar, H. S. Yathirajan, A. N. Mayekar, Maciej Kubicki

**Affiliations:** a Department of Chemistry, Adam Mickiewicz University, Grunwaldzka 6, 60-780 Poznań, Poland; b Department of Studies in Chemistry, University of Mysore, Manasagangotri, Mysore 570 006, India; c Sequent Scientific Ltd, New Mangalore 575 011, India

## Abstract

In the crystal structure of the title compound, C_10_H_8_N_4_·H_2_O, the organic mol­ecules are approximately planar [maximum deviation from the least-squares plane = 0.041 (2) Å]. Two mol­ecules are connected by two water mol­ecules *via* O—H⋯N hydrogen bonding into dimers, which are located around centres of inversion. In the crystal, mol­ecules are stacked in the *a-*axis direction, with mean distances between the π systems of 3.43 (1) and 3.46 (1) Å [centroid–centroid distances are 3.604 (2) and 3.591 (2) Å].

## Related literature

For general background to phthalazines, see: Cheng *et al.* (1999 ▶); Coates (1999 ▶); De Stevens (1981 ▶); Shubin *et al.* (2004 ▶); Tarzia *et al.* (1989 ▶); Yatani *et al.* (2001 ▶). For related structures, see: Boulanger *et al.* (1991 ▶); Burton-Pye *et al.* (2005 ▶); Zimmer *et al.* (1995 ▶). For a description of the Cambridge Structural Database, see: Allen (2002 ▶). 
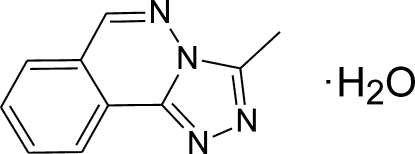


## Experimental

### 

#### Crystal data

C_10_H_8_N_4_·H_2_O
*M* *_r_* = 202.22
Triclinic, 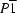

*a* = 7.3009 (9) Å
*b* = 7.9253 (9) Å
*c* = 9.2755 (10) Å
α = 109.663 (10)°
β = 104.91 (1)°
γ = 95.830 (9)°
*V* = 477.83 (10) Å^3^
*Z* = 2
Cu *K*α radiation
μ = 0.80 mm^−1^
*T* = 295 K
0.4 × 0.2 × 0.1 mm

#### Data collection

Oxford Diffraction SuperNova (single source at offset) Atlas diffractometer
Absorption correction: multi-scan (*CrysAlis Pro*; Oxford Diffraction, 2009 ▶) *T*_min_ = 0.831, *T*_max_ = 0.932
2893 measured reflections
1772 independent reflections
1615 reflections with *I* > 2σ(*I*)
*R*_int_ = 0.024

#### Refinement

*R*[*F* ^2^ > 2σ(*F* ^2^)] = 0.057
*wR*(*F* ^2^) = 0.147
*S* = 1.12
1772 reflections
164 parameters
H atoms treated by a mixture of independent and constrained refinement
Δρ_max_ = 0.20 e Å^−3^
Δρ_min_ = −0.17 e Å^−3^

### 

Data collection: *CrysAlis Pro* (Oxford Diffraction, 2009 ▶); cell refinement: *CrysAlis Pro*; data reduction: *CrysAlis Pro*; program(s) used to solve structure: *SIR92* (Altomare *et al.*, 1993 ▶); program(s) used to refine structure: *SHELXL97* (Sheldrick, 2008 ▶); molecular graphics: *XP* (Siemens, 1989 ▶); software used to prepare material for publication: *SHELXL97*.

## Supplementary Material

Crystal structure: contains datablocks I, global. DOI: 10.1107/S1600536809040677/nc2160sup1.cif

Structure factors: contains datablocks I. DOI: 10.1107/S1600536809040677/nc2160Isup2.hkl

Additional supplementary materials:  crystallographic information; 3D view; checkCIF report

## Figures and Tables

**Table 1:** Hydrogen-bond geometry (Å, °)

*D*—H⋯*A*	*D*—H	H⋯*A*	*D*⋯*A*	*D*—H⋯*A*
O1*W*—H1*W*2⋯N1	0.92 (5)	2.08 (5)	2.987 (2)	168 (4)
O1*W*—H1*W*1⋯N2^i^	0.83 (3)	2.21 (3)	3.043 (2)	177 (3)

Symmetry code: (i) 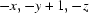.

**Fig. 1.**
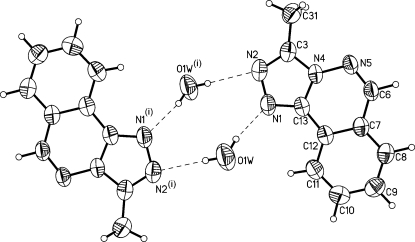
View of the dimers in the crystal structure of the title compound with labelling and displacement ellipsoids drawn at 50% probability level. Hydrogen atoms are depicted as spheres with arbitrary radii and hydrogen bonding is shown as dashed lines. Symmetry code: (i) -x,1 - y,-z.

**Fig. 2.**
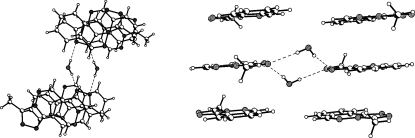
Two mutually perpendicular views of the stacking in the crystal structure of the title compound.

## supplementary crystallographic information

### Comment

The practical interest upon phthalazine derivatives is based on their widespread applications (Coates, 1999). They are commonly used as ligands in transition metal catalysis (*e.g.*, Yatani *et al.*, 2001), as chemiluminescent materials (Shubin *et al.*, 2004) and for optical applications (Cheng *et al.*, 1999). The chemistry of phthalazine derivatives has been of increasing interest since many of these compounds have found chemotherapeutic applications, especially as antihypertensive agents (De Stevens, 1981). 3-substituted 1,2,4-triazolo[3,4-*a*]phthalazines have been described as high affinity ligands for benzodiazepine receptor site (*e.g.*, Tarzia *et al.*, 1989).

In the Cambridge Database (Allen, 2002; ver. 5.30, November 2008) there are only four structures with 1,2,4-triazolo[3,4-*a*]phthalazine units. This includes 3-chloromethyl-1,2,4-triazolophthalazine (Burton-Pye *et al.*, 2005), 3-(*p*-methoxyphenyl)triazolo(4,3 - a)phthalazine (Boulanger *et al.*, 1991), 3-(*p*-methoxyphenyl)-6-(*N*,*N*-bis(2-methoxyethyl)amino)triazolo(4,3 - a)phthalazine (Boulanger *et al.*, 1991), and 3-butyl-s-triazolo(3,4 - a)phthalazine (Zimmer *et al.*, 1995). Here we present the X-ray structural analysis of the hydrate of 3-methyl[1,2,4]triazolo[3,4-*a*]phthalazine.

The molecules are almost planar with maximum deviation from the least-squares plane through all 13 ring atoms of 0.041 (2) Å (Fig. 1). The dihedral angle between the planes of the phenyl and the 1,2,4-triazole rings amount to 2.84 (7)°.

In the crystal the principal motif is built of two molecules of 3-methyl[1,2,4]triazolo[3,4-*a*]phthalazine and two water molecules, which are connected by means of O—H···N hydrogen bonds into centrosymmetric dimers which can be described according to the graph set notation as *R*_4_^4^(10), *cf.* (Fig. 2). The planar molecules are stacked in a head-to-tail manner in the direction of the crystallographic a-axis with distances between the subsequent mean planes of 3.43 (1)Å and 3.46 (1)Å.

### Experimental

1-Hydrazinophthalazine (2 g, 12.5 mmol) in 10 ml acetic acid was refluxed for 4 h. The reaction mixture was quenched to ice cold water and the solid separated was collected by filteration. The solid obtained was crystallized from methanol. Crystals for *x*-ray measurements were grown by a slow evaporation of ethyl acetate solution (m.p.: 421–423 K).

### Refinement

Hydrogen atoms from the methyl group were placed in idealized positions and were refined as riding model with *U*_iso_ values set at 1.5 times *U*_eq_ of their carrier carbon atom. All other hydrogen atoms, including those from water molecule, were freely refined.

### Crystal data

**Table:**

C_10_H_8_N_4_·H_2_O	*Z* = 2
*M**_r_* = 202.22	*F*(000) = 212
Triclinic, *P*1	*D*_x_ = 1.405 Mg m^−^^3^
Hall symbol: -P 1	Cu *K*α radiation, λ = 1.5418 Å
*a* = 7.3009 (9) Å	Cell parameters from 2647 reflections
*b* = 7.9253 (9) Å	θ = 6.4–75.6°
*c* = 9.2755 (10) Å	µ = 0.80 mm^−^^1^
α = 109.663 (10)°	*T* = 295 K
β = 104.91 (1)°	Block, colourless
γ = 95.830 (9)°	0.4 × 0.2 × 0.1 mm
*V* = 477.83 (10) Å^3^	

### Data collection

**Table:**

Oxford Diffraction SuperNova (single source at offset) Atlas diffractometer	1772 independent reflections
Radiation source: Nova (Cu) X-ray Source	1615 reflections with *I* > 2σ(*I*)
mirror	*R*_int_ = 0.024
Detector resolution: 5.2679 pixels mm^-1^	θ_max_ = 70.0°, θ_min_ = 6.4°
ω scans	*h* = −7→8
Absorption correction: multi-scan (*CrysAlis PRO*; Oxford Diffraction, 2009)	*k* = −9→9
*T*_min_ = 0.831, *T*_max_ = 0.932	*l* = −10→11
2893 measured reflections	

### Refinement

**Table:**

Refinement on *F*^2^	Primary atom site location: structure-invariant direct methods
Least-squares matrix: full	Secondary atom site location: difference Fourier map
*R*[*F*^2^ > 2σ(*F*^2^)] = 0.057	Hydrogen site location: inferred from neighbouring sites
*wR*(*F*^2^) = 0.147	H atoms treated by a mixture of independent and constrained refinement
*S* = 1.12	*w* = 1/[σ^2^(*F*_o_^2^) + (0.07*P*)^2^ + 0.128*P*] where *P* = (*F*_o_^2^ + 2*F*_c_^2^)/3
1772 reflections	(Δ/σ)_max_ < 0.001
164 parameters	Δρ_max_ = 0.20 e Å^−^^3^
0 restraints	Δρ_min_ = −0.16 e Å^−^^3^

### Special details

**Table:**

Geometry. All e.s.d.'s (except the e.s.d. in the dihedral angle between two l.s. planes) are estimated using the full covariance matrix. The cell e.s.d.'s are taken into account individually in the estimation of e.s.d.'s in distances, angles and torsion angles; correlations between e.s.d.'s in cell parameters are only used when they are defined by crystal symmetry. An approximate (isotropic) treatment of cell e.s.d.'s is used for estimating e.s.d.'s involving l.s. planes.
Refinement. Refinement of *F*^2^ against ALL reflections. The weighted *R*-factor *wR* and goodness of fit *S* are based on *F*^2^, conventional *R*-factors *R* are based on *F*, with *F* set to zero for negative *F*^2^. The threshold expression of *F*^2^ > σ(*F*^2^) is used only for calculating *R*-factors(gt) *etc*. and is not relevant to the choice of reflections for refinement. *R*-factors based on *F*^2^ are statistically about twice as large as those based on *F*, and *R*- factors based on ALL data will be even larger.

### Fractional atomic coordinates and isotropic or equivalent isotropic displacement parameters (Å^2^)

**Table:**

	*x*	*y*	*z*	*U*_iso_*/*U*_eq_	
N1	0.0820 (2)	0.4413 (2)	0.25290 (18)	0.0576 (5)	
N2	−0.0116 (2)	0.2599 (2)	0.16838 (18)	0.0585 (5)	
C3	0.0258 (2)	0.1699 (3)	0.2642 (2)	0.0504 (4)	
C31	−0.0377 (3)	−0.0275 (3)	0.2252 (2)	0.0629 (5)	
H31A	−0.1301	−0.0825	0.1193	0.094*	
H31B	−0.0966	−0.0436	0.3024	0.094*	
H31C	0.0722	−0.0847	0.2287	0.094*	
N4	0.1420 (2)	0.2902 (2)	0.41193 (16)	0.0455 (4)	
N5	0.2127 (2)	0.2489 (2)	0.54647 (17)	0.0530 (4)	
C6	0.3218 (3)	0.3879 (3)	0.6698 (2)	0.0533 (5)	
H6	0.372 (3)	0.360 (3)	0.765 (3)	0.070 (6)*	
C7	0.3736 (2)	0.5716 (2)	0.6764 (2)	0.0477 (4)	
C8	0.4993 (3)	0.7118 (3)	0.8151 (2)	0.0562 (5)	
H8	0.557 (4)	0.683 (3)	0.913 (3)	0.080 (7)*	
C9	0.5466 (3)	0.8835 (3)	0.8153 (3)	0.0600 (5)	
H9	0.636 (3)	0.978 (3)	0.909 (3)	0.067 (6)*	
C10	0.4682 (3)	0.9207 (3)	0.6786 (3)	0.0613 (5)	
H10	0.506 (4)	1.043 (4)	0.685 (3)	0.080 (7)*	
C11	0.3434 (3)	0.7866 (3)	0.5412 (2)	0.0573 (5)	
H11	0.283 (3)	0.805 (3)	0.447 (3)	0.074 (7)*	
C12	0.2970 (2)	0.6096 (2)	0.5382 (2)	0.0467 (4)	
C13	0.1741 (2)	0.4575 (3)	0.4000 (2)	0.0466 (4)	
O1W	0.2725 (3)	0.6362 (3)	0.0877 (2)	0.0876 (6)	
H1W2	0.198 (6)	0.571 (6)	0.125 (5)	0.143 (14)*	
H1W1	0.197 (4)	0.662 (4)	0.018 (4)	0.092 (9)*	

### Atomic displacement parameters (Å^2^)

**Table:**

	*U*^11^	*U*^22^	*U*^33^	*U*^12^	*U*^13^	*U*^23^
N1	0.0568 (9)	0.0734 (11)	0.0449 (8)	0.0125 (8)	0.0080 (7)	0.0313 (8)
N2	0.0558 (9)	0.0759 (11)	0.0431 (8)	0.0143 (8)	0.0092 (6)	0.0263 (8)
C3	0.0470 (9)	0.0637 (11)	0.0399 (9)	0.0137 (8)	0.0129 (7)	0.0185 (8)
C31	0.0655 (12)	0.0629 (12)	0.0510 (10)	0.0102 (9)	0.0132 (9)	0.0145 (9)
N4	0.0483 (8)	0.0565 (8)	0.0363 (7)	0.0158 (6)	0.0120 (6)	0.0228 (6)
N5	0.0653 (9)	0.0569 (9)	0.0416 (8)	0.0159 (7)	0.0124 (7)	0.0265 (7)
C6	0.0634 (11)	0.0610 (11)	0.0382 (9)	0.0159 (8)	0.0103 (8)	0.0253 (8)
C7	0.0495 (9)	0.0572 (10)	0.0413 (9)	0.0157 (7)	0.0159 (7)	0.0218 (8)
C8	0.0594 (11)	0.0645 (11)	0.0429 (9)	0.0142 (9)	0.0142 (8)	0.0191 (8)
C9	0.0565 (11)	0.0608 (11)	0.0555 (11)	0.0067 (9)	0.0189 (9)	0.0136 (9)
C10	0.0616 (11)	0.0558 (11)	0.0717 (13)	0.0122 (9)	0.0251 (10)	0.0274 (10)
C11	0.0586 (11)	0.0634 (11)	0.0599 (11)	0.0164 (8)	0.0184 (9)	0.0345 (9)
C12	0.0444 (8)	0.0585 (10)	0.0451 (9)	0.0165 (7)	0.0168 (7)	0.0255 (8)
C13	0.0454 (8)	0.0595 (10)	0.0435 (9)	0.0164 (7)	0.0150 (7)	0.0275 (8)
O1W	0.0682 (10)	0.1226 (15)	0.0838 (12)	0.0115 (9)	0.0052 (8)	0.0695 (12)

### Geometric parameters (Å, °)

**Table:**

N1—C13	1.312 (2)	C7—C8	1.398 (3)
N1—N2	1.384 (2)	C7—C12	1.404 (2)
N2—C3	1.309 (2)	C8—C9	1.369 (3)
C3—N4	1.365 (2)	C8—H8	1.01 (3)
C3—C31	1.476 (3)	C9—C10	1.391 (3)
C31—H31A	0.9600	C9—H9	0.96 (2)
C31—H31B	0.9600	C10—C11	1.370 (3)
C31—H31C	0.9600	C10—H10	0.96 (3)
N4—C13	1.369 (2)	C11—C12	1.399 (3)
N4—N5	1.3818 (18)	C11—H11	0.94 (3)
N5—C6	1.289 (2)	C12—C13	1.432 (3)
C6—C7	1.443 (3)	O1W—H1W2	0.92 (5)
C6—H6	0.97 (2)	O1W—H1W1	0.83 (3)
			
C13—N1—N2	107.24 (15)	C12—C7—C6	118.75 (16)
C3—N2—N1	108.84 (14)	C9—C8—C7	120.13 (18)
N2—C3—N4	108.18 (16)	C9—C8—H8	121.1 (14)
N2—C3—C31	127.98 (17)	C7—C8—H8	118.7 (14)
N4—C3—C31	123.82 (16)	C8—C9—C10	120.41 (19)
C3—C31—H31A	109.5	C8—C9—H9	119.8 (14)
C3—C31—H31B	109.5	C10—C9—H9	119.8 (14)
H31A—C31—H31B	109.5	C11—C10—C9	120.85 (19)
C3—C31—H31C	109.5	C11—C10—H10	122.1 (15)
H31A—C31—H31C	109.5	C9—C10—H10	117.0 (15)
H31B—C31—H31C	109.5	C10—C11—C12	119.35 (18)
C3—N4—C13	106.81 (14)	C10—C11—H11	124.5 (15)
C3—N4—N5	126.01 (15)	C12—C11—H11	116.2 (15)
C13—N4—N5	127.18 (15)	C11—C12—C7	120.10 (18)
C6—N5—N4	113.22 (14)	C11—C12—C13	124.20 (16)
N5—C6—C7	126.56 (16)	C7—C12—C13	115.70 (16)
N5—C6—H6	113.6 (14)	N1—C13—N4	108.93 (16)
C7—C6—H6	119.8 (14)	N1—C13—C12	132.48 (17)
C8—C7—C12	119.14 (17)	N4—C13—C12	118.57 (15)
C8—C7—C6	122.10 (16)	H1W2—O1W—H1W1	107 (3)
			
C13—N1—N2—C3	−0.4 (2)	C10—C11—C12—C7	1.7 (3)
N1—N2—C3—N4	0.5 (2)	C10—C11—C12—C13	−177.70 (16)
N1—N2—C3—C31	−177.91 (17)	C8—C7—C12—C11	−1.3 (3)
N2—C3—N4—C13	−0.45 (19)	C6—C7—C12—C11	179.58 (15)
C31—C3—N4—C13	178.04 (16)	C8—C7—C12—C13	178.19 (14)
N2—C3—N4—N5	179.64 (14)	C6—C7—C12—C13	−0.9 (2)
C31—C3—N4—N5	−1.9 (3)	N2—N1—C13—N4	0.06 (19)
C3—N4—N5—C6	178.98 (16)	N2—N1—C13—C12	178.52 (17)
C13—N4—N5—C6	−0.9 (2)	C3—N4—C13—N1	0.23 (19)
N4—N5—C6—C7	−0.6 (3)	N5—N4—C13—N1	−179.86 (14)
N5—C6—C7—C8	−177.53 (18)	C3—N4—C13—C12	−178.47 (13)
N5—C6—C7—C12	1.6 (3)	N5—N4—C13—C12	1.4 (3)
C12—C7—C8—C9	0.0 (3)	C11—C12—C13—N1	0.7 (3)
C6—C7—C8—C9	179.10 (17)	C7—C12—C13—N1	−178.71 (17)
C7—C8—C9—C10	0.8 (3)	C11—C12—C13—N4	179.08 (15)
C8—C9—C10—C11	−0.4 (3)	C7—C12—C13—N4	−0.4 (2)
C9—C10—C11—C12	−0.9 (3)		

### Hydrogen-bond geometry (Å, °)

**Table:**

*D*—H···*A*	*D*—H	H···*A*	*D*···*A*	*D*—H···*A*
O1W—H1W2···N1	0.92 (5)	2.08 (5)	2.987 (2)	168 (4)
O1W—H1W1···N2^i^	0.83 (3)	2.21 (3)	3.043 (2)	177 (3)

Symmetry codes: (i) −*x*, −*y*+1, −*z*.
